# Barriers and facilitators to health care seeking behaviours in pregnancy in rural communities of southern Mozambique

**DOI:** 10.1186/s12978-016-0141-0

**Published:** 2016-06-08

**Authors:** Khátia Munguambe, Helena Boene, Marianne Vidler, Cassimo Bique, Diane Sawchuck, Tabassum Firoz, Prestige Tatenda Makanga, Rahat Qureshi, Eusébio Macete, Clara Menéndez, Peter von Dadelszen, Esperança Sevene

**Affiliations:** Centro de Investigação em Saúde da Manhiça (CISM), Manhiça, Mozambique; Department of Obstetrics and Gynaecology, and the Child and Family Research Unit, University of British Columbia, Vancouver, British Columbia Canada; Ministério da Saúde, Maputo, Mozambique; Universidade Eduardo Mondlane, Faculdade de Medicina, Maputo, Mozambique; Barcelona Institute for Global Health (ISGlobal), Barcelona, Spain; Division of Women and Child Health, Aga Khan University, Karachi, Sindh Pakistan; Department of Geography, Simon Fraser University, Burnaby, British Columbia Canada

**Keywords:** Care-seeking, Maternal health, Mozambique, Prenatal care, Pregnancy, Maternal health services, Busca de cuidados de saúde, Saúde materna, Moçambique, Cuidados pré-natais, Gravidez, Serviços de saúde materna

## Abstract

**Background:**

In countries, such as Mozambique, where maternal mortality remains high, the greatest contribution of mortality comes from the poor and vulnerable communities, who frequently reside in remote and rural areas with limited access to health care services. This study aimed to understand women’s health care seeking practices during pregnancy, taking into account the underlying social, cultural and structural barriers to accessing timely appropriate care in Maputo and Gaza Provinces, southern Mozambique.

**Methods:**

This ethnographic study collected data through in-depth interviews and focus group discussions with women of reproductive age, including pregnant women, as well as household-level decision makers (partners, mothers and mothers-in-law), traditional healers, matrons, and primary health care providers. Data was analysed thematically using NVivo 10.

**Results:**

Antenatal care was sought at the heath facility for the purpose of opening the antenatal record. Women without antenatal cards feared mistreatment during labour. Antenatal care was also sought to resolve discomforts, such as headaches, flu-like symptoms, body pain and backache. However, partners and husbands considered lower abdominal pain as the only symptom requiring care and discouraged women from revealing their pregnancy early in gestation. Health care providers for pregnant women often included those at the health facility, matrons, elders, traditional birth attendants, and community health workers. Although seeking care from traditional healers was discouraged during the antenatal period, they did provide services during pregnancy and after delivery. Besides household-level decision-makers, matrons, community health workers, and neighbours were key actors in the referral of pregnant women. The decision-making process may be delayed and particularly complex if an emergency occurs in their absence. Limited access to transport and money makes the decision-making process to seek care at the health facility even more complex.

**Conclusions:**

Women do seek antenatal care at health facilities, despite the presence of other health care providers in the community. There are important factors that prevent timely care-seeking for obstetric emergencies and delivery. Unfamiliarity with warning signs, especially among partners, discouragement from revealing pregnancy early in gestation, complex and untimely decision-making processes, fear of mistreatment by health-care providers, lack of transport and financial constraints were the most commonly cited barriers. Women of reproductive age would benefit from community saving schemes for transport and medication, which in turn would improve their birth preparedness and emergency readiness; in addition, pregnancy follow-up should include key family members, and community-based health care providers should encourage prompt referrals to health facilities, when appropriate.

**Trial registration:**

NCT01911494

**Electronic supplementary material:**

The online version of this article (doi:10.1186/s12978-016-0141-0) contains supplementary material, which is available to authorized users.

## Resumo

**Antecedentes:**

Em países como Moçambique onde a mortalidade materna permanece alta, os membros de comunidades pobres e vulneráveis, residentes em zonas rurais e remotas com acesso limitado a serviços de saúde são os que mais contribuem para esta mortalidade. O presente estudo, teve como objectivo analisar comportamentos de busca de cuidados de saúde durante a gravidez, tomando em consideração as barreiras sociais, culturais e estruturais para o acesso atempado a cuidados apropriados de saúde nas províncias de Maputo e Gaza, no Sul de Moçambique.

**Métodos:**

Neste estudo etnográfico os dados foram colhidos através de entrevistas em profundidade e discussões em grupos focais com mulheres em idade reprodutiva (incluíndo mulheres grávidas), decisores ao nível do agregado familiar (parceiros, mães e sogras de mulheres grávidas), praticantes de medicina tradicional (PMTs), matronas e provedores de cuidados de saúde primários. Os dados foram analisados tematicamente usando NVivo 10.

**Resultados:**

A consulta pré-natal (CPN) era procurada pelas mulheres com o fim de abrirem a ficha pré-natal, pois mulheres sem ficha temiam represálias durante o parto na unidade sanitária. A CPN era também procurada para resolver queixas comuns tais como dores de cabeça, sintomas sugestivos de gripe, dores no corpo e de coluna. No entanto, os parceiros das mulheres grávidas consideravam as dores no baixo-ventre como o único sintoma merecedor de busca de cuidados e desencorajavam as suas mulheres a revelarem a gravidez nas primeiras semanas de gestação. Os provedores de cuidados saúde para mulheres grávidas incluíam os profissionais de saúde baseados nas unidades sanitárias, mas também matronas, anciãs, parteiras tradicionais e agentes polivalentes elementares (APES). Culturalmente as mulheres grávidas são desencorajadas a buscar cuidados junto a PMTs; no entanto observou-se que PMTs prestam serviços pré- e pós- natais. Para além dos decisores ao nível do agregado familiar, as matronas, os APEs e vizinhos são actores chaves no acto de referir a mulher grávida para a unidade sanitária. O processo de tomada de decisão envolve muitos actores e pode ser moroso e complexo, particularmente em circunstâncias de emergência em que nenhum dos decisores mencionados está presente. O acesso limitado ao transporte e a fundos para custear despesas imediatas torna este processo ainda mais complexo.

**Conclusões:**

As mulheres procuram cuidados pré natais de rotina nas unidades sanitárias, apesar da presença de outros provedores de saúde na comunidade. Existem factores importantes que impedem a busca de cuidados atempados e apropriados no início da gestação, bem como em situações de emergência obstétrica e parto. Fraco conhecimento dos sinais de perigo, particularmente entre os parceiros, a revelação tardia da gravidez, os processos complexos e morosos de tomada de decisão em situações de emergência, receio de maus tratos na unidade sanitária, falta de transporte e constrangimentos financeiros foram as barreiras mais mencionadas. Mulheres em idade reprodutiva poderiam beneficiar de esquemas comunitários de poupanças para transporte e medicação, que por sua vez iriam melhorar o seu grau de preparação para emergências e parto; mais ainda o seguimento da gravidez deveria envolver actores chaves no agregado familiar e vizinhança, bem como provedores de saúde baseados na comunidade, no sentido de encorajar referências atempadas à unidade sanitária sempre que necessário.

## Background

Maternal mortality and morbidity remain matters of public health concern. It is estimated that 303,000 maternal deaths will occur worldwide by the end of 2015. Unsafe abortions, maternal haemorrhage, and hypertensive disorders of pregnancy collectively account for nearly 50 % of all maternal deaths [1].

Although globally, there has been a 43 % decline in maternal mortality between 1990 and 2015 [2, 3], sub-Saharan Africa still contributes 62 % of maternal deaths [2]. In most cases, the highest mortality rates cluster among the marginalized and poor, who frequently reside in remote and rural areas with limited access to health care services [4].

In Mozambique, the latest maternal mortality ratio (MMR) estimates range from 249–480 per 100,000 live births [2]. Studies conducted in Mozambique indicate that haemorrhage, eclampsia, sepsis, uterine rupture and cerebral malaria are associated with the highest number of mortalities [5, 6]. Important achievements have been made with regards to antenatal care (ANC) attendance, as over 90 % of women reported having received at least one ANC visit, with slightly higher proportions in urban compared to rural areas [7, 8]. However, only around 60 % of deliveries occur at health facilities, with marked differences across regions [7, 8]. Furthermore, the magnitude of unmet need for emergency obstetric care is yet to be comprehensively addressed and is not well documented [6, 9].

In Mozambique, policies have been implemented to improve maternal and neonatal health, such as those targeting anaemia and malnutrition, the prevention of malaria in pregnancy, increased institutional deliveries, delayed age of first pregnancy, and a reduction in unsafe abortions [10]. Along with these, increasing the coverage of skilled birth attendance and ensuring resources for emergency obstetric care are urgent interventions [1]. Government programs and health strategies attempt to put such policies into practice; however, their success equally depends on the support from pregnant women and their communities. Policy recommendations must consider current behaviours, as well as the barriers and facilitators to desired care-seeking practices.

The present paper addresses the high rates of maternal morbidity and mortality related to inappropriate care seeking practices. These problematic practices include, but are not limited to, delaying the first ANC visit, associated with late disclosure of pregnancy [11, 12], as well as not meeting the minimum recommended number of ANC visits, delayed decisions to seek care for complications, and decisions not to seek skilled assistance for complications or delivery.

Factors influencing such practices, documented elsewhere particularly in Sub-Saharan Africa, include fears of medical procedures, negative attitudes of health providers [13], perceived unavailability of medication, insufficiently trained staff, and poorly equipped facilities [11, 13]. From the users’ perspective, the sudden onset of labour or the short-duration of labour, especially at night, combined with long distances influence the choice of home deliveries by traditional birth attendants (TBA) or family members [14, 15]. Local myths and misconceptions about pregnancy and birth have been noted as factors deterring health care seeking [13, 15]. Specifically, some authors argue that care-seeking during pregnancy is highly influenced by the perception adverse outcomes result from witchcraft [12].

The aim of this article is to provide insights into the understanding of women’s health care seeking practices during pregnancy, taking into account the underlying social, cultural and structural barriers to accessing timely appropriate care in Maputo and Gaza Provinces, southern Mozambique.

## Methods

### Study design

This article is part of a larger formative research study conducted in Mozambique, India, Nigeria and Pakistan, in preparation to a cluster randomized controlled trial of a Community Level Intervention for Pre-eclampsia and Eclampsia (the CLIP trial) (NCT01911494) [16].

While the formative research was based on a mixed methods approach, the present article focuses on the qualitative component conducted within an ethnographic framework [17, 18]. A detailed description of these methods is presented elsewhere [19].

### Study setting and participants

**Fig. 1:**
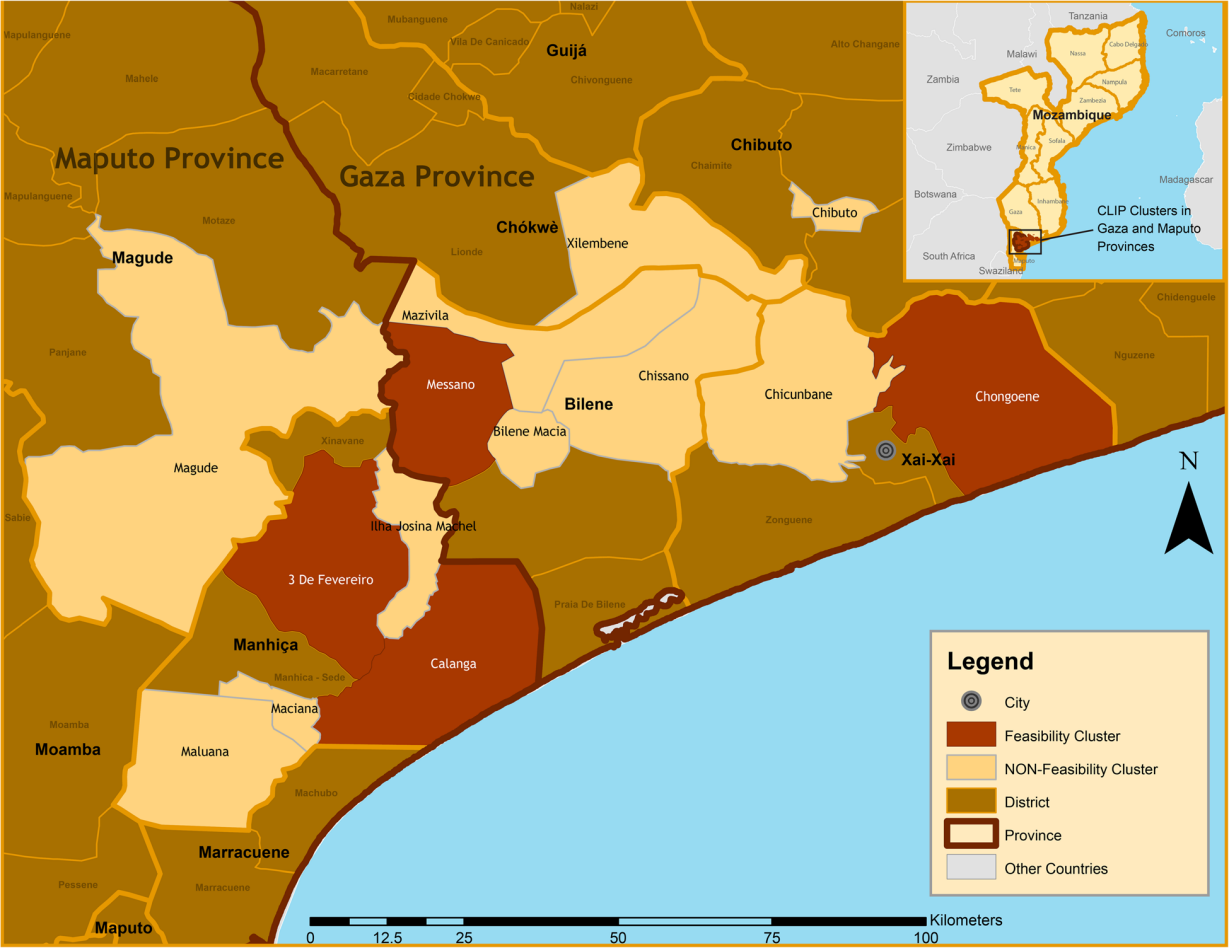
Map of the study area

The study site consisted of five Administrative Posts (APs) within three districts in southern Mozambique: Xai-Xai and Bilene-Macia districts (in Gaza Province), and Manhiça district (in Maputo Province) (Fig. 1). These APs were purposely selected to reflect the diversity of socioeconomic and demographic characteristics in southern Mozambique, such as level of urbanization, population density, distance to a trading centre, presence of referral health facilities, and physical access to them. Each of the districts and respective APs included in this study is briefly described below.

Xai-Xai, the capital town of Gaza Province, is located on the eastern coast, covering an area of 1,908 Km^2^, with a population estimated at 127,351 inhabitants [20]. The AP selected within Xai-Xai was Chongoene, which is a coastal region located 18 Km north-east of Xai-Xai. It has a population of 101,752 served by 8 primary health centres (PHC), with a total of 9 maternal-and-child health (MCH) nurses. In addition, there is access to the referral Provincial Hospital of Xai-Xai, a tertiary level facility. At the time the study was conducted, the area was also covered by 5 community health workers (CHW), locally named *Agentes Polivalentes Elementares* (APEs). Chongoene is the newly appointed head office of the district, and as result commerce, tourism, agriculture, and administrative services are thriving.

Bilene-Macia is located in southern Gaza Province, with an area of 2.180 Km^2^ and a population of 151,548 [20]. Within this district, the AP of Messano was selected for this study. It has a population of 21,471 inhabitants, and is served by two PHC with two MCH nurses and four CHWs. The referral facility is Bilene-Macia Health Centre, a secondary level facility. The community infrastructure within Messano is weak, marked by poor access to the main road. The population is primarily employed in small-scale farming.

Manhiça district is located in northern Maputo Province, 80 Km from Mozambique’s capital city. The district has an area of 2,689 Km^2^, a population of 245,829, and a mixture of urban and peri-urban communities [20]. The entire district population participates in the Health and Demographic Surveillance System (HDSS), in place since 1996 [21]. Due to the socioeconomic diversity found in this district, three APs, namely Três de Fevereiro, Ilha Josina Machel, and Calanga were selected for this study. Três de Fevereiro, located 31 Km north of Manhiça village, has 40,208 inhabitants. Four PHC with seven MCH nurses and three midwives serve the area, which had no CHWs at the time the study was conducted. Most residents are employed by the sugar and rice industry and engaged in informal trade and migrant labour in South Africa. This AP is intersected by the country’s 1^st^ National Road. It has reasonable communication networks, roads, and public services. Ilha Josina is an island 50 Km north of Manhiça Village. It has a population of 9,346 inhabitants, mostly engaged in agriculture, served by one PHC with two MCH nurse and two CHWs. Calanga is a coastal AP, located 25 Km east of Manhiça Village, with a population of 9,524 inhabitants (mostly fishermen and small-scale farmers) served by one PHC with one MCH nurse and five CHWs. Both Ilha Josina Machel and Calanga are characterized by poor road infrastructure and transportation networks, severely affected by harsh weather conditions during the rainy season. Manhiça District Hospital is a referral facility for these three APs, although some patients from Ilha Josina Machel can also be sent to Xinavane Rural Hospital. Both hospitals are secondary level facilities.

The study participants comprised of community members and health care providers. Community members consisted in women of reproductive age between 18 and 49 years (including pregnant women), male and female decision-makers (elders, husbands, partners, mothers, and mothers-in-law of women of reproductive age). Health care providers included formally-recognized cadres within the national health services (nurses, midwives, medical technicians) and traditional health care providers (TBAs, matrons and traditional healers). Although traditional health care providers in Mozambique often have interchangeable roles [22], it is worth mentioning some important differences among them. Traditional healers are mostly sought for the diagnosis, treatment and protection from illness, misfortune and other social concerns [23]; TBAs provide assistance to women during pregnancy, birth and the postpartum period; matrons are responsible for performing a variety of rituals including those for new-borns and adolescents [22, 24].

### Data collection and analysis

Data collection consisted of focus group discussions (FGD) and individual in-depth interviews (IDI) conducted with community members and health care providers (Tables 1 and 2). Focus groups and individual interviews were chosen to gain an understanding of the social norms and local contexts underlying care seeking practices, rather than the individual experiences and meanings assigned to them. Interviews were conducted when it was not practical to convene the required number of participants within a specific target group.

**Table 1:** Focus group discussions conducted

FGD target group	Number of FGDs	Number of participants
Women of reproductive age	6	35
Mothers and mothers-in-law	7	51
Partners, husbands, and other male decision makers	8	39
PHC nurses, midwives, and assistant medical officers	7	25
Matrons and traditional birth attendants	5	46
TOTAL	33	196

**Table 2:** In-depth interviews conducted

Target group	Number of IDIs
District medical officers	3
Traditional healers	4
Traditional birth attendants	1
Elders	5
TOTAL	13

The number of focus groups and individual interviews was pre-determined based on previous experiences of reaching data saturation regarding similar topics in different contexts [25, 26]. Both across and within-group saturation was assessed. For interviews with health care providers, the snowball sampling approach was used for recruitment, drawing initially on existing networks of local investigators and health professionals at the sites. Community members were identified through community leaders, who were provided with the required socio-demographic characteristics for inclusion. Focus groups were usually conducted out-doors at the community’s “circle” (location of community leaders’ office); individual interviews were conducted at the participants house.

Interviews and focus groups were conducted by trained facilitators belonging to the social science research unit of the Manhiça Health Research Centre (CISM). Gender balance within the team members was ensured to cater for possible gender-sensitive issues, especially in one-on-one interviews.

All interviews and focus groups, of which some were conducted in Portuguese and others in Changana (local dialect) according to participants’ preference, were audio recorded, and transcribed verbatim in Portuguese, preferably by the same team members who collected the data. Data quality checks were done by the social science team leader by reviewing the transcripts while listening to the audio recording. Data was analysed using NVivo version 10.0 (QSR International Pty. Ltd. 2012). Thematic data analysis was performed through the following steps: generating categories, coding text according to each category; annotating emerging themes and patterns and readjusting the categories and relationships between them; testing emergent themes through systematic searches of coded text; investigating alternative explanations through systematic searches of uncoded text. The social science team leader and the study’s senior social scientist conducted the coding of all Portuguese transcripts at CISM. Assistance was given from the CLIP social science co-ordinator from the University of British Columbia (UBC), who spent a significant amount of time onsite to support training, oversee data collection and perform the analysis quality control through repeat coding of one third of transcripts, which were translated into English, and evaluating coding agreement.

All data collection was conducted after obtaining signed informed consent from each participant, as well as permission to record individual interviews and group discussions. Ethical approval for this study was obtained from the CISM Institutional Review Board (CIBS – CISM) and the UBC Review Board.

## Results

The results of the overall formative research are presented in Additional file 1. The findings of this qualitative analysis include a detailed account of routine and emergency care-seeking practices during pregnancy, as well as health care seeking behaviours for delivery and postpartum. Following this, participants describe the complexities of decision-making at the household and neighbourhood-level. Finally, study results identify the social, economic and structural factors that affect the decision and ability to seek timely and appropriate care during pregnancy, complications, delivery and postpartum.

### Interview and focus group discussion respondents

The core data for this study component was generated from 33 FGDs involving 196 participants, which included women of reproductive age (WRA), mothers, mothers-in-law, and partners of WRA, nurses, midwives, medical assistants, matrons, and TBAs. Additionally, the study included 13 IDIs with district medical officers, traditional healers, elders, and one TBA who was not able to attend the FGDs. Tables 1, 2, 3 and 4 provide detailed information regarding the number and characteristics of participants from each target group.

**Table 3:** Characteristics of FGD participants

Characteristic	*N* (%)
FGD target group	
Women of reproductive age	35 (18)
Mothers and mothers-in-law	51 (26)
Partners and husbands	39 (20)
PHC workers	25 (13)
Matrons and traditional birth attendants	46 (23)
Age	
18–25	35 (18)
26–39	53 (27)
40–59	46 (23)
≥ 60	44 (22)
Unknown	18 (9)
Gender	
Female	148 (76)
Male	48 (24)
Marital status	
Single	32 (16)
Married	119 (61)
Separated/divorced	5 (5)
Widow(er)	25 (13)
Unknown	15 (8)
Education level	
No formal education	47 (24)
Primary level 1 (grade 1–5)	82 (42)
Primary level 2 (grade 6–7)	21 (11)
Secondary level (grade 8–10)	14 (2)
Pre-university level	14 (2)
Unknown	18 (9)
Main occupation	
Housewife/Unemployed	22 (11)
Subsistence farmer	118 (60)
Self-employed/Small business	5 (3)
Formally employed (health sector)	26 (13)
Formally employed (other sector)	10 (5)
Unknown	15 (8)
Religion	
Christian (Zionist)	83 (42)
Christian (Assembly of God)	25 (13)
Christian (Catholic)	25 (13)
Other Christian	27 (14)
Atheist/Animist	8 (4)
Unknown	48 (24)

**Table 4:** Characteristics of IDI participants

Characteristic	*N* (%)
Age	
18–25	0 (0)
26–39	5 (38)
40–59	2 (15)
≥ 60	5 (38)
Unknown	1 (8)
Gender	
Female	9 (69)
Male	4 (31)
Marital status	
Married	8 (62)
Single	2 (15)
Widow(er)	3 (23)
Education level	
No formal education	2 (15)
Primary level	8 (62)
Secondary/Pre-university level	0 (0)
Higher level	3 (23)
Main occupation	
Traditional healer	4 (31)
Community elder	6 (3)
Medical doctor	3 (23)

### When and where do women seek care during pregnancy, delivery and post-partum?

#### Routine antenatal care to guarantee a healthy pregnancy

Pregnant women had the unanimous view that the health facility was the appropriate place to seek care during pregnancy, alleging that they had not heard of any alternative location. When probed about the circumstances under which women sought such care, “opening the antenatal record” (the act that marks the first ANC visit) and “attending mothers’ weighing day” (weight monitoring visits) were the key events mentioned.

Health professionals revealed that it was common practice, however, to attend the first ANC visit after completion of the first trimester.

*“They come late [to the first ANC visit], usually on the fourth month. There is a misconception that it is only appropriate to come to the health facility when the pregnancy shows or when the baby starts moving”* (Health professional, Bilene-Macia)

Male decision-makers revealed that traditional medicines were available to everybody in the community, including to pregnant women, but they were not regularly used during pregnancy. This was further described by mothers and mothers-in-law, who stated that even though traditional medicines might help pregnant women, they may kill the baby. Nevertheless, some pregnant women claimed to take traditional medicines (herbal mixtures soaked in water) to treat minor ailments such as stretch marks. Elders advised pregnant women to use traditional methods to guarantee positive pregnancy outcomes; this concept is known as “closing the pregnancy”, and is intended to prevent miscarriages and premature births.

*“What takes us to give traditional medicine is when there is a risky pregnancy, and here we “close it”. We look for those traditional remedies so that the pregnancy does not suffer, then we boil them and give to her to drink in very small amounts, and then tie the remainders together, because they are roots and not leaves, and insert them into a jar or a ximbitana (small clay pot) which is buried in a place…so the pregnancy and the unborn child are healthy. The day the baby wants to come out, we go to the place and unbury the jar, as soon as we unbury it the baby is out”* (Mothers and mothers-in-law, Calanga)

#### Care seeking in case of illnesses or complications

Some women described that they accessed health services only in the advent of illness. The ailments mentioned by pregnant women as triggers for treatment seeking at the ANC clinic were headaches, flu-like symptoms, perceived high blood pressure, body pain and backache. Participants also mentioned that care was sought when they realised that the illness was serious.

*“It is the diseases, if you go to the hospital it is because you are sick”* (Women of reproductive age, Ilha Josina Machel)

Many participants identified “activists”, which likely referred to CHWs, as the first point of contact for complications in pregnancy. CHWs were reported to help pregnant women to reach the health facility. It was also mentioned that in many emergency situations, pregnant women were assisted by TBAs and other community members, who based their actions on local knowledge. In cases of seizures and loss of consciousness, for example, community members claimed to revive women by blowing on her face, dropping cold water in her ears, or exposing her to strong smells (including shoes or crushed aromatic leaves) before taking her to a health facility.

Ultimately, participants seemed to highly value the role of the health facility in resolving complications if care was sought promptly.

*“You feel dizzy, strong palpitations, you lose your senses and stop recognizing where you are…if you are not close to the hospital you may lose your life. If you are close to the hospital, the doctors can observe you quickly and you can recover.”* (Pregnant women, Calanga)

Even traditional healers expressed that they needed information regarding health facility diagnosis. *“When they [pregnant women] come here, I first search in my tinlholo (stones and bones) to find out what’s in her body. When it seems like it has nothing to do with us [tradition]…I tell her: “go to hospital to be examined”, and then she comes back and I do what has to be done”* (Traditional healer, Chongoene)

#### Care seeking for delivery and postpartum

Women felt that using health care services for delivery was imperative. Pregnant women also identified particular conditions during delivery which require care urgently, such as breech presentation.

*“Because at the hospital they know that the baby is not well positioned and they know what to do in order to return the baby to the normal position”* (Pregnant women, Três de Fevereiro)

According to FGDs, men and matrons considered the presence of abdominal pain and labour as the most important reasons to go to the health facility.

Despite the recognition that the hospital was crucial, hesitation of pregnant women to seek assistance at the health facility was sensed from discussions. Fear of mistreatment by health care providers was a recurrent reason presented by pregnant women (Table 5). They were particularly concerned about the treatment given to women without antenatal records or those who gave birth outside the health facility.

**Table 5:** Issues and concerns regarding TSB by stakeholder group and study area

	Study area (administrative post)				
Target group	Messano	Ilha Josina	Chongoene	Calanga	Três de Fevereiro
Pregnant women	- Partners slow/or inadequate response to emergency - Partners inability to recognize warning signs requiring EmOC	- Weather conditions deteriorate quality of roads	- Long distances between homes and health facilities - Lack of access to transport - Partners decide on different care providers (e.g.: faith healers)	- Partners do not help with child caretaking and domestic chores - Distance between homes and health facilities - Lack of transportation - Scarcity of CHW - Intimidating attitudes of health facility - Women’s inability to meet the costs of care in the hospital - Medication stock-out	- Lack of ambulances
Male partners	- Lack of transport to the main road - Lack of money for transport - No-one to care for the children and household chores in the absence of the pregnant women	- Partners inability to recognize warning signs - Transporters do not accept to carry severe cases - PHC facility unable to deal with complications	- Local health facility not prepared to assist complications - Lack of ambulance for swift referrals - Lack of money for transport	- Lack of money for transport - Partners do not feel empowered to assist pregnant women	- Lack of ambulances - Lack of money to pay for transport - Pregnant women’s physical vulnerability prohibitive of walking - Unclear price list for the few cars that are available
Mothers, mothers in law of WRA	- Partners slow/or inadequate response to emergency - Lack of money to support transportation expenses	- Pregnant women keep going to the cultivating fields, increasing the risk of being alone and helpless when emergency occurs	- Limited number of CHWs - High cost of transport - Women fear going to the hospital	- Negative attitudes of health professionals	- No-one helps pregnant women at home - Difficulties in requesting a lift to those who have vehicles
Elders	- Pregnant women not satisfied with the results of the previous treatment received at the health facility - Nurse not always present at the health facility - Medication stock outs - Habit of seeking traditional treatment first - Delays in seeking care	- Not mentioned	- Inability to meet consultation cost (coupon) - Pregnant women do not follow hospital recommendations - Tradition “hides” the real diagnosis - Lack of transport to reach the main road - Small health facility not prepared for emergencies	- Not mentioned	- The habit of seeking traditional medication first
TBAs/Matrons	- Lack of transport within the neighborhood - Lack of money to pay for public transport on the main road	- Lack of income sources to pay for health care expenses in general - Men’s lack interest in taking up child caretaking and domestic chores	- Negative attitude of health professionals - Fear that health professionals will find out about previous traditional treatments taken	- Long distances between homes and health facilities - Lack of transport in the area - Medication stock-outs - Men prioritizing pleasure (ex: purchasing alcohol) over pregnancy wellbeing	- Polygamous partners not interested in the pregnancy follow-up - Lack of adequate transport for sandy roads
Health workers	- Partners’ lack of interest in “females’ issues” - Communication barriers among couples (due to HIV sero-status) - Delays in seeking ANC care	- Delays in seeking care in general	- Delays in seeking assisted delivery - Poor quality of roads	- Delays in seeking care in general - Lack of transport	- Delays in seeking care in general

*“Going to the hospital is a norm because you cannot give birth at home. If you leave home while you are having labour pains and give birth on the way and arrive at the hospital with the child in your hands, they are going to ask you: ‘How did you manage to give birth on the way? Why did you not go to the hospital?’ They shout at you and leave you with your baby”* (Pregnant women, Três de Fevereiro)

Another concern, which pregnant women believed the health professionals disregarded, is the lack of transportation.

*“They [the health professionals] tell us off because sometimes you arrive….here in our community there is a scarcity of transport and you arrive late. And when you enter [the health facility] first they shout and only then they assist us, although you were late not because you wanted to, it was because of the transport to arrive…here it is far.”* (Pregnant women, Chongoene)

When discussing about the issue of delays with health professionals, they only attributed this to negative attitudes of the pregnant women, not accounting for other factors, mentioned by pregnant women, that may prevent them from seeking prompt and appropriate assistance.

*“I tell some [women] during their pregnancy that “your delivery will be at the provincial hospital”, then comes the moment of pain and she remains at home. Because I had told her that she had to prepare herself to be near the doctor, she prefers to stay at home, and when it complicates it is when she tries to run here.”* (Health professionals, Chongoene)

Matrons have been discouraged from assisting deliveries at home. The fear of infectious diseases, such as HIV, has increased concordance with this mandate.

*“If you see that she is on her days of giving birth, you must quickly take her to hospital. There you will only find midwives from the hospital, not from outside the hospital because there is AIDS, you will have contact with her while she is not healthy and then touch the blood of those sensitive areas. You will then get the disease. Even if she is your daughter you must take her to hospital” (Matrons and TBAs, Três de Fevereiro)*

Male participants described circumstances when deliveries were unavoidably assisted by matrons or elders in the community. In those cases, postpartum care was sought immediately after birth with the main objective of ensuring good health for the baby.

*“It may happen that the day of giving birth comes and she is not able to go to the hospital…there are elders that can help her give birth. And after the birth they take the child to the hospital”* (Male decision-makers, Três de Fevereiro)

Even in circumstances of unavoidable delivery outside a health facility, there were fears of the repercussions, as expressed by mothers/mothers-in-law.

*“Even if we help someone to give birth, you help her but when you take her to hospital, you arrive there and they pull your ears for having done that woman’s labour…you will be to blame because you are the “community nurse”, and they wouldn’t even insult that woman who refused to go to hospital, but you who helped her…and they send you both back home”* (Mothers and mothers-in-law, Calanga)

Traditional healing was described as playing a role after delivery; however, respondents were less concerned about the risks of traditional medicine to the baby after delivery. Postpartum care for mother and baby was based on home remedies prescribed by elders. One mention of traditional treatment consisted of leaves soaked in water, which is believed to help breastfeeding for first time mothers.

*“When you are starting to have children, to avoid passing your days which can result in a dead baby, they find the leaves and soak them in water and then you start taking them. They also do that to “accelerate” the breastfeeding because when the baby suckles the nipples are torn…so that treatment kills the bug that tears the nipples”* (Mothers and mothers-in-law, Calanga)

### What are the decision-making patterns for care seeking in pregnancy, delivery and postpartum?

The decision to seek care varied according to the circumstances under which care was needed. Under usual circumstances, pregnant women themselves decided whether and when to attend the first antenatal visit as well as subsequent routine visits. However, pregnant women underlined that partners should be informed of their decision and they are expected to obtain permission.

*“You decide personally once you see that these months are enough for me to open a file. You tell him that today is a new day and I am going to the hospital to open a file. And he says: “go”. Because you have to go there every month”* (Women of reproductive age, Três de Fevereiro)

The main circumstance in which partners discouraged pregnant women from seeking antenatal care was when they considered the pregnancy to be “too small to be disclosed”. This was emphasised by participants in Messano and Ilha Josina Machel.

In case of illness, complications, or delivery, pregnant women were less likely to act as the primary decision-makers; as a result, care seeking was frequently delayed by the partners decision-making. At times, delays resulted from the perception that it is inappropriate to seek care too early in pregnancy.

*“Some husbands may see that she [the wife] is vomiting too much and tell her to go to hospital, but others may leave you [the wife], alleging that it [the belly] is still small, while in the end, you will lose your strength and lose water”* (Women of reproductive age, Messano)

In other cases, the delays were linked to the husband’s financial constraints.

*“If your belly aches you are the one who feels it. If you tell him and he doesn’t answer you as he should say… you have to go because you know where the hospital is. He says that he is still looking for money.”* (Mothers and mothers-in-law, Messano)

Finally, delays in seeking care for complications occurred when husbands or household decision-makers were unaware of the warning signs of pregnancy.

*“If a person who knows these things has the knowledge that in that house there is a serious illness, if one goes to visit and realises that they are not taking her to hospital, they can ask if she is taking medication. If they say that she is not, then the visitor calls the parents or the owners of the house and tell them to take to the hospital”* (Partners and husbands, Ilha Josina Machel)

According to matrons and TBAs, although the husband was the primary decision-maker, in practice he was usually not around when complications occurred. In such circumstances, mothers-in-law made decisions regarding care.

*“It is the mother…Yes, if she is still alive. Because the husband will be busy committing adultery”* (Matrons and TBAs, Três de Fevereiro)

Husbands also claimed that neighbours could substitute them in the decision-making process, and they particularly favoured female accompaniers. Only under exceptional circumstances, husbands and male partners considered the possibility of being more actively involved in the process.

*“It depends on our union….we can help each other as neighbours, but because this is mothers’ work …I cannot go and take his wife [pointing to another man]. As a man, I cannot because it [labour or complications] may start on the way… now if it is a mother, yes, they are going to become birth attendants on the way and they are going to support that person until the baby is out, and they are going know how to take her to the hospital.”* (Male partners and husbands, Calanga)

Matrons and TBAs were the only entities, besides immediate family members and neighbours, with the authority to make decisions regarding care seeking for pregnancy complications, especially when there was no time or means to locate other decision-makers. In Calanga, where people live dispersedly and have considerable challenges accessing transport and communication, efforts were made to identify neighbours who informed the family that a woman was being taken to the health facility.

Significant importance was placed on accompaniment and support from the husband, other family members or neighbours; however, these support systems may be lacking for some. Single women and adolescents had more difficulty accessing services in pregnancy, as they had less support. Particularly in Três de Fevereiro, participants stated that the rejections of pregnancies by partners was quite common, resulting in single mothers, often young girls, living with their mothers who act as the primary decision maker.

### What are the barriers to care seeking for pregnant women?

Although ANC and delivery services are free of charge, indirect costs, primarily from transport and medication, were strong barriers to care seeking among pregnant women. Medication was said to be particularly costly if it was not available at health facilities, leading patients to resort to private pharmacies. Traditional treatments were stated to be more expensive because of the charges associated with consultations and medications, even though there was no need for transport, as traditional healers were widely available in the community.

Most women in these areas did not have their own source of income, nor did they participate in community savings schemes such as *xitique*, an informal rotating savings and credit association, serving as a form of mutual assistance among women, relatives, close friends or co-workers, consisting in a collective periodical contribution of a fixed amount of money, which is assigned in turn to each member [27]. Therefore, pregnant women were financially dependent on their partners. As mentioned earlier, when partners were not able to provide financially, then treatment was delayed or not sought at all. Some pregnant women said that in extreme cases they did odd jobs (such as cultivating land plots) to earn money to buy medications from private pharmacies or to pay for traditional treatments. This strategy was most commonly used for traditional care, because the need for funds was not immediate, as expressed by some pregnant women in Calanga.

*“Yes, and if I go there [traditional healer] and say that I am ill he will not deny me the remedy just because I do not have money, he will give me the remedy and when I get better I will find money to pay”* (Women of reproductive age, Calanga)

Access to the health facilities was limited by transport availability. In some communities, there was no transport available to assist pregnant women to health facilities as the road conditions were poor while in other communities the options were prohibitively expensive.

*“Access to roads, exactly that is what I was saying; roads, that can affect a pregnant woman”* (Health professionals, Chongoene)

Some women lived at great distances from the health facilities, which provided an additional challenge. Even in places where transport was readily available (mostly mini-buses or agriculture tractors), it was restricted to main roads; therefore, many residents must walk long distances to reach transport. Weather conditions further hampered women’s ability to access health services in many regions.

*“When there is rain, these are no conditions for walking”* (Women of reproductive age, Ilha Josina Machel)

Discussions regarding limited access to health services match with the local belief that links illness and misfortune.

*“Pregnancy complications bring us misfortunes because we don’t have transport that can take us, in order to take her to hospital. People are really dying. Do you see all this sandy area, for you to push a wheelbarrow? … So what use does it have? Even a truck has no use here”* (Matrons and TBAs, Três de Fevereiro)

Health workers and community members alike complained about the lack of ambulances; they are only available to transport patients from health facility to health facility. Those with access to ambulances are precisely those who have already been privileged to be able to reach the health facility.

*“Regarding transport even being at the hospital, once you say that we need an ambulance the day can end before getting it”* (Partners, Três de Fevereiro).

Women requiring admission to hospital were concerned about the need to arrange for the caring of their small children and household chores in their absence. Additionally, household members must provide meals for pregnant or postpartum women admitted at the health facility.

*“We really need it [money] because since you are going to Manhiça Hospital, knowing that you do not have family there, you have nothing there, anything [food] you want you have to buy”* (Pregnant women Calanga)

The above situation highlights the many household- and community-level factors contributing to delays in accessing health services.

## Discussion

This study examines the patterns of care seeking by pregnant women in communities of Maputo and Gaza Provinces, southern Mozambique, with a particular focus on antenatal care, emergency obstetric care, delivery and the postpartum period. A variety of perspectives were captured including those from pregnant women, key decision-makers, and care providers.

The data generated from this study demonstrated that in these communities women patronize a range of care providers, from the ANC clinic to traditional healers, traditional birth attendants, matrons, elders, as well as community health workers when available. This diverse set of providers reflects a typical scenario of medical pluralism described in many contexts worldwide, with the strong presence of two prominent systems of health care provision in Africa, namely biomedicine and ethnomedicine [28, 29]. The type of provider sought depends on the perceived aetiology of the condition, as well as the circumstances of the woman at that moment. It is this set of circumstances that is often not taken into account when efforts to improve care-seeking behaviours are made. This was evident in this study through the health professionals’ unawareness of community factors related to inappropriate care-seeking practices.

The hospital was identified by participants as the most appropriate place for pregnant women to seek care. Even the alternative care providers (such as matrons, TBAs, elders and traditional healers) reported referring their patients to health facilities. The preference for care at health facilities, which has been found in other studies in Mozambique [30, 31], was prominent when related to opening the antenatal record or delivery.

Of note, because partners discouraged women from revealing their pregnancy early in gestation, women tended to attend the first ANC visit quite late. Results from other studies in Mozambique attribute this as an act of protection of the pregnancy from witchcraft [11, 12]. This in itself constitutes a missed opportunity for care.

Some argue that antenatal care is an important determinant of safe delivery and overall maternal and new-born health [32, 33]; however, findings from this study illustrate that the determinants of accessing routine ANC may differ from those associated with emergency care or delivery. Therefore the opportunity to use antenatal check-ups to identify women at risk of complications may be missed if the unique context underlying care-seeking is not addressed.

The findings from this study demonstrate a commitment, by formal and informal health care providers, patients and community members alike, to promote ANC and facility delivery. Nevertheless, more should be done both at community and at health system levels to ensure prompt care seeking for early and regular ANC, mild ailments, obstetric complications, delivery and postpartum care. Consistent with existing evidence, these findings suggest a need for improving community knowledge about the danger signs of pregnancy [34, 35]; yet, this alone is insufficient and needs to be accompanied by interventions aligned with existing social and cultural constructions of pregnancy, birth, and postpartum [13].

Despite the evidence that pregnant women are discouraged from taking traditional medicines, this study suggests that this is a reflection of cultural norms, to which there are exceptions. Participants of this study reported use of traditional remedies for the treatment of some ailments and to prevent adverse pregnancy outcomes such as miscarriages, premature births, and stillbirths. Such practices were also reported during postpartum. Traditional health care providers (matrons, elders, TBAs and traditional healers) should not be ignored as important entry points for the identification and referral of pregnant women at risk [10, 14].

Decision-making processes regarding care-seeking in pregnancy are complex and reflect women’s social and economic conditions. Importantly, it is precisely the cases requiring prompt and effective treatment (early gestation, complications and delivery) which encompass the most complex decision-making processes. In these circumstances, pregnant women have little to say regarding when and where to seek care. Their fate is determined by the decision makers’ capacity to recognise warning signs, their financial aptitude, priority settings, and preferences towards the available care providers. Similar results were obtained in Tanzania, where husbands and elders were identified as the decision-makers for reproductive health matters, yet they were rarely the target groups of reproductive health interventions [36]. As a result, as has been shown in this study, decision-makers remain unaware of the danger signs of pregnancy, resulting in no progress in terms of reducing delays in seeking care.

Young mothers are a particularly vulnerable group with an MMR up to 1.5 times higher than mothers over 19 years of age [5]. The present study has shown that this particular group is highly affected by the complex decision-making patterns in these communities, since they are more likely to live with their mothers due to the rejection by the baby’s father. Special attention must be paid to improve the interactions with this group and with their mothers as decision-makers.

Long distances to health facilities and poor access to transport were important factors raised both in this study and elsewhere [14]. Extreme weather conditions regularly deteriorated the quality of already precarious roads, making access increasingly difficult in the rainy season. Community-based health providers, such as TBAs, matrons and CHWs are geographically more accessible; however, there is no evidence that they are prepared to assist or refer obstetric emergencies, apart from TBAs in some areas of Mozambique [30, 31]. Even among TBAs, policy and interventions have been geared towards some technical skills, such as recognition of warning signs and immediate care for the new-born [37]. The importance of referral is also stressed, but it is not sufficiently addressed from the broader ecological perspective, that is, taking into account the socio-cultural context [38].

This study illustrated that despite free health care in pregnancy, indirect costs, which included transportation, medication, and household-level logistics required for hospital admission, were often prohibitive. By comparing the dynamics of traditional treatments with care at health facilities, it can be argued that it is the modality of payment rather than the absolute cost which may constitute a barrier. Traditional care can incur much higher charges; however, these providers tend to allow greater flexibility of payment which is in line with local fund-raising strategies such as borrowing or engaging in odd jobs. These results also strengthen the evidence that within vulnerable communities, pregnant women tend to be among the most economically and socially marginalised [11], as shown by their inability to make independent decisions and mobilize funds to meet their own needs. There is a need for promotion of birth preparedness and complication readiness, which is the process of planning for delivery and preparing for the possibility of an obstetric emergency [39]. This approach has been shown to be successful in improving access to services in other resource constrained settings, for instance in Burkina Faso [40]. There is also a need to potentiate economic security and autonomy of pregnant women [11] as part of the birth preparedness and complication readiness arrangements, with particular attention to single women, adolescents, and women who have other children and household chores under their responsibility.

A gender analysis is recommended for future research on factors that affect health-related decisions of pregnant women in similar contexts. This approach can further speak to the role of the social and cultural context in women’s health-care decision making, by highlighting gender roles, their associated inequities, and how these impact health care decision in pregnancy and postpartum [41–43].

## Conclusions

Women regularly seek ANC at health facilities. Factors that impede women from timely accessing maternal health services include societal discouragement from revealing pregnancies early in gestation, unfamiliarity with the warning signs of pregnancy among women and their partners, complex and delayed decision-making, fundraising strategies misaligned with formal payment schemes for health care-related expenses, poor transport infrastructure, and fear of mistreatment at health facilities. Strategies are needed at the community and health system levels to improve maternal health, which are in concordance with the formal health care system structure as well as the societal networks of these communities. Women of reproductive age would benefit from saving schemes for transport to health facilities and medication. Pregnancy follow-up should include key family members, and community-based health care providers should encourage prompt referrals to health facilities.

## Additional file

Additional file 1: Peer review reports. (PDF 244 kb)

## Abbreviations

AIDS: Acquired Immunodeficiency Syndrome
ANC: antenatal care
AP: Administrative Post
APE: Agente Polivalente elementar
CHW: community health workers
CISM: Manhiça Health Research Centre
FGD: focus group discussions
HIV: human immunodeficiency virus
MMR: maternal mortality ratio
TBA: traditional birth attendants
TSB: treatment seeking behaviour
UBC: University of British Columbia
WRA: women of reproductive age
