# Supplementary material for: Barriers and facilitators to health care seeking behaviours in pregnancy in rural communities of southern Mozambique
Source: Reprod Health. 2016 Jun 8;13(Suppl 1):31. doi: 10.1186/s12978-016-0141-0 (PMC4943506; doi:10.1186/s12978-016-0141-0)
Supplement: Additional file 1: — Peer review reports. (PDF 244 kb) [file 12978_2016_141_MOESM1_ESM.pdf]

Referee's comments to the authors– this sheet WILL be seen by the author(s) and published with the article

Title	**Barriers and facilitators to health care seeking behaviours in pregnancy in rural communities of southern Mozambique**
Author(s)	Khátia Munguambe, Helena Boene, Marianne Vidler, Cassimo Bique, Diane Sawchuck, Tabassum Firoz, Prestige Tatenda Makanga, Rahat Qureshi, Eusébio Macete, Clara Menendez, Peter von Dadelszen, Esperança Sevene
Referee's name	Bukola Fawole

When assessing the work, please consider the following points, where applicable:

1. Is the question posed by the authors new and well defined?
2. Are the methods appropriate and well described, and are sufficient details provided to replicate the work?
3. Are the data sound and well controlled?
4. Does the manuscript adhere to the relevant standards for reporting and data deposition?
5. Are the discussion and conclusions well balanced and adequately supported by the data?
6. Do the title and abstract accurately convey what has been found?
7. Is the writing acceptable?

Please make your report as constructive and detailed as possible in your comments so that authors have the opportunity to overcome any serious deficiencies that you find and please also divide your comments into the following categories:

- Major Compulsory Revisions (which the author must respond to before a decision on publication can be reached)
- Minor Essential Revisions (such as missing labels on figures, or the wrong use of a term, which the author can be trusted to correct)
- Discretionary Revisions (which are recommendations for improvement but which the author can choose to ignore)

Where possible please supply references to substantiate your comments.

When referring to the manuscript please provide specific page and paragraph citations where appropriate.

General comments:
Authors have employed qualitative design to investigate the perceptions of women, family members, important decision makers, community leaders, health care providers in the formal and informal sectors regarding health seeking behavior during pregnancy, delivery and post partum period.

They identified certain socio-cultural norms, ignorance, infrastructural and economic factors as important influences and barriers to health seeking behavior in the rural community of Mozambique.

Major compulsory revisions:

None

Minor essential revisions:

The manuscript will benefit from some copy editing. For example, the expression 'attendance to first ANC visits' requires rephrasing.

(continue on the next sheet)

Continued
Readers will probably be keen to know the number of interviews conducted particularly when Authors report that issues were exhausted to the point of saturation. It will be helpful to provide this within the Results section.

Authors state that they recorded the consent process. This requires clarification. Does this imply that the recording process started prior to obtaining informed consent from the participants?

Who are ‘matrons’?

Several of the quotations were attributed to groups of participants. One would have expected that individual quotes are to be are to be reported representing particular themes..:

Referee's comments to the authors– this sheet WILL be seen by the author(s) and published with the article

Title	**Barriers and facilitators to health care seeking behaviours in pregnancy in rural communities of southern Mozambique**
Author(s)	Khátia Munguambe, Helena Boene, Marianne Vidler, Cassimo Bique, Diane Sawchuck, Tabassum Firoz, Prestige Tatenda Makanga, Rahat Qureshi, Eusébio Macete, Clara Menendez, Peter von Dadelszen, Esperança Sevene
Referee's name	Neale Smith

When assessing the work, please consider the following points, where applicable:

1. Is the question posed by the authors new and well defined?
2. Are the methods appropriate and well described, and are sufficient details provided to replicate the work?
3. Are the data sound and well controlled?
4. Does the manuscript adhere to the relevant standards for reporting and data deposition?
5. Are the discussion and conclusions well balanced and adequately supported by the data?
6. Do the title and abstract accurately convey what has been found?
7. Is the writing acceptable?

Please make your report as constructive and detailed as possible in your comments so that authors have the opportunity to overcome any serious deficiencies that you find and please also divide your comments into the following categories:

- Major Compulsory Revisions (which the author must respond to before a decision on publication can be reached)
- Minor Essential Revisions (such as missing labels on figures, or the wrong use of a term, which the author can be trusted to correct)
- Discretionary Revisions (which are recommendations for improvement but which the author can choose to ignore)

Where possible please supply references to substantiate your comments.

When referring to the manuscript please provide specific page and paragraph citations where appropriate.

General comments:

This paper reports on factors that affect decisions of pregnant women in rural Mozambique to seek or not seek antenatal, delivery and postnatal care. It is a qualitative study, part of the larger CLIP trial. The approach is suited to the topic. As noted below, the results speak to a number of gender-related issues. A feminist or gender lens would seem quite appropriate to the project – but I appreciate that this is not the methodology selected by the authors.

Major compulsory revisions:

I would like to see more detail in parts of the methods section, for instance at line 120 where it is stated "Some data was translated to English". How was it determined which data to translate, and which not? Line 109 refers to "required sociodemographic characteristics for inclusion". I presume those are the ones described in the preceding paragraph, but this is not entirely clear. Some description of the research sites (line 95) might be helpful as well, as the relevance of this comes up later in the Results section (see my comment later). Most importantly, I would like the researchers to articulate how and why they decided upon the particular mix of interviews and focus groups used in the study. Why both? What does the mix add, or is it simply a pragmatic decision based on informants' availability? I also think the authors should provide method references for their key concepts, ethnography (line 92), and thematic data analysis (line 122).

Major compulsory revisions, *Continued*:

Tables 1 and 2 could be considerably more informative, I thought. Table 1 for instance gives the # of focus groups, but I would like also to know the total # of participants. And given the nature of the results, which dwell substantially on gender-related issues (male decision making authority in the household, etc.), I would like to know the gender breakdown of respondents. It also might be helpful to the reader to know the breakdown of respondents across the three research sites (line 95), or between community members and health care providers (lines 98-103).

I do think the Results would benefit greatly from a summary table which explicitly compares similarities and differences among main groups (men, women, traditional and Western providers) in relation to the themes. I was left wondering otherwise if all the perspectives had been captured and reported. For instance, at lines 196 and 206, it is reported that the formal health system itself places barriers in the way of women seeking care; do the providers interviewed concur with this? Similarly, at line 291 it is noted that “matrons and TBAs” report that male decision makers were “usually not around when complications occurred”. Is that validated by the male heads of household interviewed?

At line 302, the authors describe one finding that is particular to one of the sites—which leaves me to wonder if this is the only such instance where site matters to the findings, or if there are others that should also be noted.

Minor essential revisions:

I found the aim of the paper (line 81) to be a bit vague—to “describe” health seeking behaviours. While this is appropriate for qualitative research, it also comes in the context of the previous paragraph which speaks of a number of relevant factors as previously identified in the literature. I would think that the aim would at least partly also be to reflect, with these findings, on where the literature is or is not supported.

Discretionary revisions:

I thought it would be helpful to begin the Results section with a paragraph which states the main themes that will be described therein.

There are a few minor stylistic issues to be addressed, such as consistent formatting of references, occasional typo. I thought some of the phrasings were a bit lengthy and cumbersome, e.g., at line 455: “The results of this study have shown that in this setting…” Greater use of the active voice might make the writing more vibrant.

Supplement Editor comments:

Please improve the references following the style of the Journal, in some of them the year is missing, others only put the first author. In references not coming from journals the link and date that this link was accessed is needed.
